# Vocal cords palsy in systemic lupus erythematosus patient: diagnostic and therapeutic difficulties

**DOI:** 10.1007/s00296-012-2615-x

**Published:** 2012-12-25

**Authors:** Piotr Leszczynski, Katarzyna Pawlak-Bus

**Affiliations:** 1 Division of Rheumatology and Osteoporosis, Jozef Strus Hospital, 61-285 Szwajcarska 3, Poznan, Poland; 2 Department of Physiotherapy, Rheumatology and Rehabilitation, University School of Medical Sciences, Poznan, Poland

**Keywords:** Vocal cords palsy, Myasthenia, Neuropsychiatric systemic lupus erythematosus (NPSLE)

## Abstract

Vocal cords palsy is a rare complication in the course of systemic lupus erythematosus (SLE). A 38-year-old female patient with a history of SLE presented with chronic voice hoarseness resistant to standard treatment. High levels of antinuclear antibodies including dsDNA, Ro52, SSA, SSB were confirmed, while antiphospholipid antibodies were absent. While other causes of voice hoarseness were excluded, bilateral vocal cords palsy was diagnosed. Moreover, the patient revealed features of obvious Hashimoto thyroiditis with high levels of antithyroid antibodies and also developed a convergent squint as a result of fatigability of oculomotor muscles. Electrophysiology test of peripheral nerves detected myasthenic type nerve-muscle conduction impairment which was suspected as the cause of reported symptoms. Possible reasons for emerging signs and symptoms of neuropsychiatric systemic lupus erythematosus were discussed as well as the presence of vasculitis, neuropathy, significance of thyroiditis and coexistence of myasthenia. All that reasons of similar autoimmune background were also raised in this case report.

## Introduction

Systemic lupus erythematosus is an autoimmune disease with a wide range of symptoms and a variety of causes for observed abnormalities. Fluctuating activity creates a mosaic of signs and symptoms. Their diagnosis and understanding enable an effective causal treatment. Clinical picture reflects the disease activity; therefore, it outlines the course and intensity of treatment.

The interpretation of symptoms is further complicated by circulating active antibodies, exacerbation of inflammatory process, especially in blood vessels, influence of drugs, and also possibility of other autoimmune disorders co-morbidity. The range of neurological symptoms accompanying NPSLE is very wide, and their presence is associated with a poor prognosis. Neurological symptoms can be primary or secondary, and their differentiation may cause diagnostic difficulties. In the presented case, the larynx was affected which is not a common clinical manifestation of lupus, and voice hoarseness due to vocal cords palsy is rare.

## Case report

The described case is a 38-year-old female patient with a 5-year history of systemic lupus affecting skin, mucous membrane, joints, recurrent leucopenia and myocardial infarction and transient ischemic attacks. The patient has suffered from hypertension and dyslipidemia which also is present in her family members. Anti-double strand DNA (dsDNA), anti-SSA and SSB antigens as well as anti-Ro52 antibodies were demonstrated. Disease activity was maintained at a stable level (maximum <10 in the SLEDAI scale) during the treatment with hydroxychloroquine and low doses of corticosteroids. To date, apart from minimal deterioration of cognitive functions revealed in psychological tests. The patient has not demonstrated neurological symptoms.

In November 2010, the patient developed acute hoarseness resistant to antibiotic treatment. Bilateral vocal cords palsy, more prominent on the left side, was confirmed by laryngological examination. Two months later, convergent squint was noted. Infection, neoplasm, local and metabolic causes were excluded as the reason for those symptoms. Neither concomitant exacerbation of lupus symptoms nor the presence of antiphospholipid antibodies (aPL) were observed. However, it was noted that levels of anti-thyroglobulin and anti-thyroperoxidase antibodies were high. Thyroid gland was inflamed but not enlarged on ultrasound investigation, and its hormonal function was normal. Neuropathy was suspected but magnetic resonance imaging (MRI) of head and neck did not reveal features of vasculitis or other organic causes. Electromyography revealed nerve-muscle conduction impairment similar to myasthenia which could explain hoarseness as well as oculomotor muscle fatigability (VI cranial nerve). The patient received corticosteroids pulse therapy as well as immunosuppressive treatment with cyclophosphamide. Adding anticholinergic drug brought about gradual improvement, resolution of hoarseness and visual disturbances. The patient received methylprednisolone pulse therapy with cumulative dose 9 g during 3 months. In addition, cyclophosphamide treatment was given intravenously first two cycles of 600 mg dose and then of 400 mg dose in further 4 cycles. The patient received a total amount of 2,800 mg cyclophosphamide in 2–3 weeks intervals. Between corticosteroids pulse cycles therapy maintenance of 4–8 mg of methylprednisolone orally was used. Simultaneously, the patient took pyridostigmine as an anticholinergic therapy initially at dose 60 mg three times daily. The dose was gradually increased to 120 mg three times daily. Three months later, the patient was asymptomatic and after the 6-month therapy did not require maintenance doses of corticosteroids. Anticholinergic treatment was continued for the next 6 months. Then, it was discontinued without progression of muscle-nerve conduction symptoms at 1-year follow-up.

## Discussion

Despite advancement in diagnostic tools, it is difficult to attribute neurological symptoms to an autoimmune process. There is a broad variety of those symptoms (see Table 1) from discrete impairment of cognitive function, anxiety disorders, polyneuropathy to manifestations such as stroke or psychosis.

**Table 1:** Symptoms of NPSLE and its prevalence [1]

NPSLE symptoms		
Frequent >5 %	Rare 1–5 %	Extremely rare <1 %
Headaches Cognitive function impairment Mood changes Epilepsy Disease of brain vessels (stroke, TIA) Anxiety disorders	Psychoses Polyneuropathy	Cranial nerves neuropathy Mononeuropathy Aseptic meningitis Demyelination syndrome Autonomic dysfunction Myasthenia

*NPSLE* neuropsychiatric systemic lupus erythematosus

*TIA* transient ischemic attack

All those symptoms are included to American College of Rheumatology (ACR) classification criteria defining NPSLE symptoms [1]. Neurological signs may precede classical SLE as well as appear in the course of described case. Vocal cords and oculomotor muscles palsy are not a common manifestation of SLE. Irrespective of the type of neurological symptoms, involvement of the nervous system always indicates a poor prognosis. Even mild neurological impairment justifies intensification of treatment.

Can we predict the risk of development of neurological symptoms in SLE? Is there anything in clinical picture of the described case that would suggest a neurological background of reported symptoms? Currently, it is thought that both SLE-dependent risk factors for the involvement of nervous system and those factors not related to it should be taken into account. Hypertension, dyslipidemia, previous myocardial infarction and brain ischemia in such a young age intensify vigilance to symptoms of NPSLE but also increase the risk of manifestation of corticosteroid therapy complications. On the other hand, low activity of SLE, minor cognitive function impairment and lack of antiphospholipid antibodies do not increase the risk of neurological symptoms. More than half the patients with SLE suffer from NPSLE symptoms [2], and 10 years after initial diagnosis only 21 % of them retain normal cognitive functions [3].

Pathogenesis of NPSLE has been attributed to antibodies causing neural dysfunction including antiphospholipid antibodies and vasculopathy, especially microangiopathy with a dominant inflammatory process or thrombosis. Focal abnormalities in NPSLE as vasculitis, myelopathy, cranial nerve neuropathy are usually associated with thromboembolic events. Diffuse abnormalities such as psychosis are more often related to the single neurons defect due to the presence of antibodies or an inflammatory process [4]. Each of those mechanisms may play a role in the formation of neurological symptoms and most often they coexist. Some studies indicate that in patients with NPSLE, the anti-ribosomal protein P and lupus anticoagulant antibodies are found more often [5], which was not observed in our subject. Also, aPL and anti-neuronal antibodies were not detected in this patient.

Having watched the development of new neurological symptoms in the presented patient with SLE, it seems to us that the most likely SLE cause is vasculitis. Diagnostic imaging performed in this patient did not reveal changes characteristic of vasculitis which could explain peripheral neuropathy [6]. MRI of nervous system is a gold standard diagnostic tool for NPSLE. Normal results of central nervous system imaging in the presence of this clinical picture and previous vascular events are surprising. It raises question of MRI sensitivity but also the quality of radiological assessment. In the case of pathologic changes in the course of lupus, it requires an extreme precision and an experienced radiologist. The sensitivity of MRI in NPSLE is 50 % for diffuse changes and 80 % for focal abnormalities. An association between the localization of focal lesions in the central nervous system and a range of neurological symptoms in SLE has not been proved.

A turning point was the assessment of nerve-muscle conduction function and a detection of myasthenia type conduction impairment. Coexistence of myasthenia and SLE is reported in the literature but is a rare instance [7]. In prospective analysis of NPSLE symptoms involving 1,206 patients, there were no cases of myasthenia [8]. The incidence of myasthenia in SLE is 0.1 % [4]. It is difficult to be entirely sure whether myasthenia type symptoms can be attributed to NPSLE. Can pathogenesis of conduction impairment be related to an autoimmune process or is it a separate disease? Analyzing existing clinical observations, it can be concluded that more often myasthenia patients develop SLE symptoms than the other way round. The fact is that an autoimmunological process which leads to the formation of antibodies may affect many different clinical areas. This means that the coexistence of several types of antibodies and their various expressions is entirely possible and has been gracefully compared to kaleidoscope of lupus disease (“kaleidoscope phenomenon”) [9]. This is a good parallel for dynamic changes in immunological system and their multifactorial background. One of such autoimmunological conditions frequently accompanying SLE is a chronic thyroiditis which was also present in the described case [10].

In the literature, the main clinical symptom described for myasthenia disorders in SLE patients is a skeletal muscle weakness. Symptoms associated with the third and the fourth cranial nerves dysfunction are less often described [11, 12]. Cranial nerves neuropathy in the course of SLE is also rare and range between 0.5 and 1.0 % [4]. A differential diagnosis between neuropathy and myasthenia symptoms is difficult, and results are often unequivocal. Neuropathy is often caused by vasculitis (vasa nervorum) and correlates with a severe disease course, other symptoms of vasculitis and high activity of SLE [6]. Myasthenia symptoms are a result of nerve-muscle conduction dysfunction. Unfortunately, presented case was atypical. Localization of symptoms and the involvement of larynx were quite surprising. Vocal cords palsy developed without features of larynx inflammation which suggested a neurological background. In the literature, there are cases described where larynx was involved including vocal cords palsy in the course of SLE [13, 14]. However, its direct cause has not been identified.

Is myasthenia in patients affected by SLE a part of the disease course or sign of its exacerbation or a separate entity?

Activity of SLE can be assessed with SLEDAI and BILAG activity scales in an attempt to objectively measure the degree of disease severity. These scales assess symptoms and their severity, considerate additional laboratory results, and can allocate corresponding scores. There tools may be more objective assessment of the patient. Neurological symptoms of systemic lupus achieve the highest scores in SLEDAI disease activity scale. Its interpretation is therefore crucial in a diagnostic process and is often a starting point for further management.

The patient received an immunosuppressive therapy due to a neurological character of the described symptoms and their association with clinical states of lupus activity. This could improve a nerve-muscle conduction apart from the anticholinergic treatment. It is known from the literature that in the isolated cases of myasthenia, the effectiveness of the immunosuppressive therapy including cyclophosphamide and corticosteroids has been previously reported [15]. The results were positive, but no randomized clinical trials were conducted.

## Summary

Kaleidoscope of systemic lupus symptoms and its dynamic course requires an appropriate interpretation of observed symptoms and an objective assessment of the disease activity. Management of patients with NPSLE requires a great experience, diagnostic insight, detailed differentiation and therapeutic decisions according to treat-to-target goal. Primary NPSLE is reflected of disease activity with less than 40 % of neurological symptoms arising in the course of SLE. Secondary NPSLE is reflected as complications and side effects of our treatment and occurs more often. All a both could be consider in differential diagnosis of primary and secondary changes in the course of NPSLE. Myasthenia symptoms in SLE are often surprising and rare. Without identification of the underlying cause of neurological impairment, we may treat patient unsuccessfully with high risk of therapeutic complications with any clinical improvement. An immunological storm, which accompanies exacerbation of SLE and a wide range of symptoms, makes the systemic lupus, especially with nervous system involvement, a challenge both from diagnostic and from therapeutic points of view.
